# Harnessing the Regenerative Potential of Fetal Mesenchymal Stem Cells and Endothelial Colony-Forming Cells in the Biofabrication of Tissue-Engineered Vascular Grafts (TEVGs)

**DOI:** 10.1155/2024/8707377

**Published:** 2024-06-12

**Authors:** Angus Weekes, Joanna M. Wasielewska, Nigel Pinto, Jason Jenkins, Jatin Patel, Zhiyong Li, Travis J. Klein, Christoph Meinert

**Affiliations:** ^1^Centre for Biomedical Technologies, Queensland University of Technology (QUT), Brisbane, QLD, Australia; ^2^School of Mechanical, Medical and Process Engineering, Faculty of Engineering, Queensland University of Technology (QUT), Brisbane, QLD, Australia; ^3^Herston Biofabrication Institute, Metro North Hospital and Health Services, Herston, QLD, Australia; ^4^Faculty of Medicine, The University of Queensland, Brisbane, QLD, Australia; ^5^Department of Vascular Surgery, The Royal Brisbane and Women's Hospital, Herston, QLD, Australia; ^6^School of Biomedical Sciences, Faculty of Health, Queensland University of Technology (QUT), Woolloongabba, QLD, Australia

## Abstract

Tissue engineering is a promising approach for the production of small-diameter vascular grafts; however, there are limited data directly comparing the suitability of applicable cell types for vessel biofabrication. Here, we investigated the potential of adult smooth muscle cells (SMCs), placental mesenchymal stem cells (MSCs), placental endothelial colony-forming cells (ECFCs), and a combination of MSCs and ECFCs on highly porous biocompatible poly(*ɛ*-caprolactone) (PCL) scaffolds produced via melt electrowriting (MEW) for the biofabrication of tissue-engineered vascular grafts (TEVGs). Cellular attachment, proliferation, and deposition of essential extracellular matrix (ECM) components were analysed *in vitro* over four weeks. TEVGs cultured with MSCs accumulated the highest levels of collagenous components within a dense ECM, while SMCs and the coculture were more sparsely populated, ascertained via histological and immunofluorescence imaging, and biochemical assessment. Scanning electron microscopy (SEM) enabled visualisation of morphological differences in cell attachment and growth, with MSCs and SMCs infiltrating and covering scaffolds completely within the 28-day culture period. Coverage and matrix deposition by ECFCs was limited. However, ECFCs lined the ECM formed by MSCs in coculture, visualised via immunostaining. Thus, of cells investigated, placental MSCs were identified as the preferred cell source for the fabrication of tissue-engineered constructs, exhibiting extensive population of porous polymer scaffolds and production of ECM components; with the inclusion of ECFCs for luminal endothelialisation, an encouraging outcome warranting further consideration in future studies. In combination, these findings represent a substantial step toward the development of the next generation of small-diameter vascular grafts in the management of cardiovascular disease.

## 1. Introduction

The field of regenerative medicine has experienced significant growth since the biofabrication of the first tissue-engineered vascular graft (TEVG) in 1986 [1]. Comprised of a combination of fibroblasts, smooth muscle cells (SMCs), endothelial cells (ECs), and collagen, this first construct formed the basis for vascular tissue engineering for years to come. Since then, many cell sources and types have been investigated for graft biofabrication, as reviewed extensively throughout the literature [2–7]. However, very few studies have progressed beyond preclinical trials, particularly in small-diameter applications, with graft failure often attributed to inadequate biological and mechanical performance [8, 9]. While advancements in additive manufacturing and biomaterials have produced a considerable number of semisynthetic and biological TEVGs which fulfil certain requirements, a consensus is yet to be reached on the ideal combination of cell source and type for vascular regeneration [4, 10, 11].

The production and maintenance of functional vasculature are highly dependent on active cell-driven processes [4]; hence, cell types utilised *in vitro* directly dictate the structural and functional development of TEVGs [10]. Selection criteria for vascular tissue formation include the proliferative and migratory capacity of cells, particularly within biomaterial substrates; the ability for preservation of synthetic functionality; and deposition of essential extracellular matrix (ECM) components [4, 12]. The ability of applicable cell types to fulfil these requirements is highly dependent on source and phenotype, while optimised culture conditions are imperative for tissue growth. Furthermore, the availability and accessibility of cell sources, and their scalability toward clinical outcomes must also be considered [7, 11].

Physiologically, blood vessels are comprised of concentric layers exhibiting specific ECM composition, structure, and function. Elongated ECs form a luminal coating, with cells aligned longitudinally by arterial shear flow [10]. The endothelium exhibits unique antithrombogenic properties, mitigating platelet aggregation, coagulation, and inflammation, while regulating nutrient diffusion with the blood [4, 11, 13]. The tunica media is comprised primarily of circumferentially aligned SMCs, which exhibit a contractile phenotype, within an ECM abundant in elastin, proteoglycans, and collagen type I and III which ensure mechanical compliance [11]. The surrounding adventitia provides substantial strength through a complex morphology of collagen with varied fibril orientation [14].

Thus, in accordance with native vessel requirements, it is well established that engineered grafts with an endothelialised luminal surface, strengthened by a collagen and elastin-rich ECM, maintain greater patency *in vivo* [15–19]. Throughout regenerative medicine, autologous cell sources are often considered ideal for patient-specific applications given their nonimmunogenic properties [20]. However, autologous cells characteristically exhibit limited proliferative potential, insufficient for the scalable production of cultured constructs [20–23]. This is particularly limiting in the most prevalent patient demographic, whereby accessibility and availability of suitable cells are reduced [11, 24], with age-associated senescence a significant factor [25]. Furthermore, extended *in vitro* culture of adult SMCs may lead to dedifferentiation toward an activated state [26]. Thus, while many studies have utilised SMCs with positive results, characterisation of phenotype modulation is required to mitigate dedifferentiation from a quiescent state, which may cause fibrotic ECM deposition and stenosis [26].

Through advancements in regenerative medicine, many issues associated with adult cell types have been surpassed with stem cell sources, as viable alternatives for tissue engineering [11, 27, 28]. Stem cells, which possess a high capacity for self-renewal and differentiation into various cell lineages, may be isolated from embryonic or adult sources [27]. Embryonic stem cells are frequently used in research practice, though there are substantial ethical considerations that may limit upscaled production [3]. Mesenchymal stem cells (MSCs), which exhibit myogenic differentiation potential and capacity for self-renewal, have been extensively studied [27]. Gong and Niklason [29] demonstrated the capacity of bone-marrow MSCs, seeded onto poly(glycolic acid) (PGA) scaffolds, to differentiate toward a vascular phenotype. MSCs also exhibit high genomic stability and immunosuppressive capacity, in addition to lesser ethical considerations, validating their use as a viable option in biofabrication technologies [3, 27].

Endothelial progenitor cell types, such as endothelial colony-forming cells (ECFCs), represent a suitable cell type for the endothelialisation of TEVGs [27]. Exhibiting high proliferative capacity, ECFCs may be isolated from bone marrow, as well as peripheral or cord blood, with negligible differences in biological performance between sources reported in the literature [30]. However, availability may limit production scalability, with further research and development into efficient biomanufacturing processes required. Hence, the investigation into appropriate cell types and sources is required in all biofabrication approaches to vascular tissue engineering.

Herein, the findings of a comparative study investigating the applicability of commercially available adult SMCs, alongside pure fetal MSCs and ECFCs isolated with high yield from human placental tissue, cultured on biomimetic poly(*ɛ*-caprolactone) (PCL) scaffolds toward the biofabrication of TEVGs, are presented in detail. The highly compliant PCL scaffolds utilised herein were produced via melt electrowriting (MEW), based on our previous studies which comprehensively describe the development of biomimetic substates for TEVG biofabrication [31]. As a biocompatible, biodegradable polymer, medical grade PCL represents an idealised material option for use in tissue engineering studies, suitable for cell attachment as demonstrated throughout the literature [32, 33]. Furthermore, in MEW additive manufacturing, PCL remains the gold-standard material for the manufacture of microfibrous scaffolds with defined architecture, given its low melting temperature and long-term stability [34, 35]. In this study, it was hypothesised that both SMCs and MSCs, which exhibit high biological plasticity [36, 37], would proliferate extensively within the scaffold substrates, synthesising abundant ECM components [38, 39]. Furthermore, ECFCs were incorporated to ascertain suitability for luminal endothelialisation in the fabrication of TEVGs. Thus, in combination with our previously published findings into the development of biomimetic scaffolds suitable for the biofabrication of small diameter TEVGs, the outcomes of this work represent a substantial step toward successful long-term studies in the biofabrication of biologically relevant TEVGs which fulfil the physiological requirements of native blood vessels.

## 2. Materials and Methods

### 2.1. Fabrication of PCL Scaffolds

A custom-built melt electrowriting (MEW) device, described previously [40, 41], fitted with a stainless steel collector was utilised for the manufacture of tubular scaffold constructs (4.0 mm diameter and 80 mm length) (Figure S1A, Supplementary Data). Scaffold designs exhibited biomimetic architecture for physiologically relevant mechanical performance, as comprehensively characterised in our previous studies [31]. Medical grade poly (*ɛ*-caprolactone) (PCL) (Mw = 81 kg/mol, Mn = 54 kg/mol) (Purasorb PC 12, Corbion, the Netherlands) was used as received. For extrusion of molten PCL, a 3 cc syringe barrel (Nordson, USA) fitted with a 23 G general purpose tip (Nordson, USA) was heated to 80°C within the MEW print head. The temperature was stabilised for 15 min prior to printing and maintained throughout. Extrusion pressure was set to 0.025 MPa, with an electric potential of 4.0 kV for electrohydrodynamic control of the polymer jet. A distance of 4.0 mm was set between the nozzle and collector, with speed maintained at 280 mm/min. Following fabrication, scaffolds were stored in humidity-controlled conditions.

### 2.2. Cell Expansion

Commercially sourced SMCs (CC-2571, Lonza, Switzerland) were cultured in Smooth Muscle Cell Growth Medium (SmGM-2) (CC-3182, Lonza, USA) containing 0.1% (v/v) insulin, 0.2% (v/v) human fibroblastic growth factor (hFGF-B), 0.1% (v/v) gentamicin sulfate-amphotericin (GA-1000), 0.1% (v/v) human epidermal growth factor (hEGF), and 5% (v/v) fetal bovine serum (FBS). Fetal MSCs and ECFCs were isolated from full-term human villous placental tissue via published protocols established by us, with potency confirmed via phenotypic characterisation as described and presented previously [42–44]. For *in vitro* expansion of the MSC and ECFC populations, an optimised media composition has been established by the authors of [42], for the culture of these cells independently or in coculture, given their isolation from the same tissue source. Hence, MSCs and ECFCs were expanded in endothelial cell growth medium (EGM-2) (CC-3162, Lonza, USA) containing 0.04% (v/v) hydrocortisone, 0.4% (v/v) hFGF-B, 0.1% (v/v) vascular endothelial growth factor (VEGF), 0.1% (v/v) recombinant insulin-like growth factor (R3-IGF-1), 0.1% (v/v) ascorbic acid, 0.1% (v/v) hEGF, and 0.1% (v/v) GA-1000, 0.1% (v/v) heparin, supplemented with 12% (v/v) FBS as we have described [42]. Cells were cultured at 37°C, 5% CO_2_, and 95% humidity until 80% confluent, prior to scaffold seeding.

### 2.3. Scaffold Seeding

To ensure hydrophilic surface conditions, PCL scaffolds were plasma-treated (Figure S1B, Supplementary Data) with O_2_/Ar (15/5) for 2 min at 38 W in a vacuum plasma cleaner (PDC-002-HP, Harrick Plasma, USA) as validated in our work previously [31], to induce complete scaffold wettability suitable for cell seeding with limited influence on scaffold mechanics [45, 46]. Samples were then obtained using a 5 mm diameter biopsy punch (Figure S1C, Supplementary Data) and used for cell seeding within 24 hours. PCL scaffold samples were sterilized in 80% (v/v) ethanol for 30 min and allowed to dry via evaporation inside a biosafety cabinet for 1 hour. Scaffolds were seeded separately with SMCs, MSCs, ECFCs, and a coculture of MSCs + ECFCs (1 : 5 cell density), at a concentration of 10 million cells per mL, using 5 *μ*L per sample (Figures S1D–S1G, Supplementary Data). While a 1 : 1 ratio of MSCs to ECFCs was considered, in line with previous studies utilising these specific cell types in coculture [47, 48], the 1 : 5 ratio at which MSCs and ECFCs were seeded within the coculture group was determined based on the hypothesis that the MSCs would be expected to attach to, and branch across, the scaffolds at a greater proliferative rate than the ECFCs. Furthermore, given the negligible branching capacity of ECFCs and their propensity for surface layer migration, a higher density of ECFCs was used in the coculture seeding suspension in this study. Samples were placed in an incubator for 2 hours for initial attachment, with 0.5 mL of media subsequently added to each sample in 24-well plates, with exchanges made every 2 days thereafter.

### 2.4. Cell Viability

Live and dead cell imaging was performed on D1, D14, and D28 with fluorescein diacetate (FDA) (cat. no. F1303, Invitrogen, USA) and propidium iodide (PI) (cat. no. P1304MP, Invitrogen, USA) to determine cell viability. TEVG samples from each group were washed in PBS, stained in 10 *μ*g/mL FDA and 5 *μ*g/mL PI in PBS for 5 min, and rinsed with PBS. Images were obtained via fluorescence microscopy (AxioObserver Z1, Carl Zeiss, Germany), with cell viability and coverage determined from integrated fluorescence intensity measurements and stained area, respectively, via ImageJ [49]. Values were reported as mean ± SD (*n* = 3–6). It must be noted that due to the dense branching at D14 and D28, specific live and dead cell counts were unsuitable to obtain, hence the use of fluorescence intensity measures. As such, immunofluorescence staining, as described subsequently, enabled the quantification of cell nuclei to complement the semiquantitative viability data obtained via the methods described here.

### 2.5. Metabolic Activity

Analysis of metabolic activity within TEVG samples (*n* = 6) was performed on days 1, 14, and 28. Culture media was aspirated prior to the addition of 0.5 mL PrestoBlue assay reagent (1 : 10, cat no. A13261, Invitrogen, USA) in new culture media. Samples were then incubated at 37°C, 5% CO_2_, and 95% humidity for 45 minutes. Fluorescence measurements were performed with an excitation wavelength of 540 nm and emission of 590 nm (ClarioStar Plus, BMG Labtech, Germany). Values were reported as mean ± SD (*n* = 6).

### 2.6. Scanning Electron Microscopy

Images of TEVG samples were obtained across timepoints via scanning electron microscopy (SEM) (Phenom XL G2, ATA Scientific Pty Ltd, Australia) enabling characterisation of scaffold architecture, cell attachment, and coverage. Samples were fixed in 4% (w/v) paraformaldehyde (PFA) for 30 min and then washed and stored in PBS, prior to dehydration in serial ethanol dilutions and platinum sputter coating. Images were obtained via SEM at an 8 mm working distance under vacuum conditions, with an accelerating voltage of 5 kV. Fibre diameter and pore measurements were determined via ImageJ [49]. Values were reported as mean ± SD (*n* = 60).

### 2.7. Immunofluorescence

The phenotypic characterisation of SMCs, MSCs, and ECFCs within TEVGs was determined via immunostaining. Samples were fixed in 4% (w/v) PFA for 30 min at room temperature and then washed and stored in PBS. Prior to staining, samples were incubated in blocking and permeabilization buffer containing 5% (v/v) donkey serum (cat. no. D9663, Sigma-Aldrich, USA) and 0.1% (v/v) Triton X-100 (cat. no. X100, Sigma-Aldrich, USA) in PBS at 4°C overnight. Primary antibodies against *α*-SMA (1 : 100, cat. no. 7817, Abcam, UK), CD90 (1 : 100, cat. no. MA5-32559, Invitrogen, USA), and CD31 (1 : 50, cat. no. 561653, BD Biosciences, USA) were used, respectively, for staining of SMCs, MSCs, and ECFCs, applied overnight at 4°C. Subsequently, samples were incubated with donkey antimouse Alexa Fluor 488 (1 : 500, cat. no. A32766, Invitrogen, USA), donkey antirabbit Alexa Fluor 555 (1 : 500, cat. no. A32794, Invitrogen, USA), and Alexa Fluor 633 phalloidin (1 : 500, cat. no. A22284, Invitrogen, USA), respectively. Samples were counterstained with 4,6-diamidino-2-phenylindole (DAPI) (5 µg/mL, cat. no. D1306, Invitrogen, USA) to visualise cell nuclei and imaged via confocal laser scanning microscopy (CLSM) (TCSSP5 Confocal, Leica Microsystems, Germany). Stained nuclei counts were obtained to complement viability measures obtained, with DAPI-stained images binarized and analysed in ImageJ [49]. Values were reported as mean ± SD (*n* = 3–6).

### 2.8. DNA and GAG Quantification

To quantify retained DNA and glycosaminoglycan (GAG) content across timepoints, TEVG samples (*n* = 6) were frozen on days 1, 14, and 28. Samples were digested overnight in phosphate-buffered EDTA (PBE) containing 0.5 mg/mL proteinase K (cat. no. 25530015 Invitrogen, USA) at 55°C on a thermomixer (Eppendorf, Germany). DNA concentrations in the digests were determined using the Quant-iT PicoGreen dsDNA quantification assay (cat. no. P7589, Invitrogen, USA). Furthermore, a dimethyl-methylene blue (DMMB) (cat. no. 341088, Sigma-Aldrich, USA) assay was used to determine GAG content in digests, with absorbance measured at 525 and 595 nm (ClarioStar Plus, BMG Labtech, Germany). Concentrations were calculated from the ratio of absorbances and compared to a chondroitin sulfate (CS) standard (cat no. C4384, Sigma-Aldrich, USA). Values were reported as mean ± SD (*n* = 6).

### 2.9. Histology

TEVG samples from D1, D14, and D28 fixed in 4% (w/v) PFA and stored in PBS were embedded in 2% (w/v) agarose gel (Meridian Life Science, USA) for immobilisation during tissue processing (Leica 300S, Leica Microsystems, Germany). Samples were embedded in paraffin, with 5 *μ*m sections collected for staining and imaging analysis. Separate sections from each group were stained with hematoxylin and eosin (H&E) to visualise nuclei distribution and ECM deposition, Masson's trichrome (MTC) stain for collagen and muscle components, and van Gieson's (VG) stain for collagen and elastin components, using standard protocols. Slides were imaged via brightfield microscopy (AxioObserver Z1, Carl Zeiss, Germany).

### 2.10. Collagen Staining

For immunofluorescence imaging of collagen I and III, 5 *μ*m sections were first subject to antigen retrieval using 0.1% (w/v) hyaluronidase (cat. no. H3506, Sigma-Aldrich, USA) in PBS at 37°C for 30 min. Primary antibodies against collagen I (1 : 200, cat. no. PA1-26204, Invitrogen, USA) and collagen III (1 : 500, cat. no. PA5-34787, Invitrogen, USA) were applied overnight at 4°C, after blocking in 5% (v/v) donkey serum (cat. no. D9663, Sigma-Aldrich, USA) in PBS overnight at 4°C. Donkey antirabbit Alexa Fluor 488 (1 : 500, cat. no. A32790, Invitrogen, USA) was then applied overnight at 4°C and then counterstained with DAPI (5 *μ*g/mL, cat. no. D1306, Invitrogen, USA). The absence of nonspecific staining was verified using negative controls stained without the application of primary antibodies. Sections were imaged via fluorescence microscopy (AxioObserver Z1, Carl Zeiss, Germany). For quantification, integrated fluorescence intensity measurements of collagen I and III were obtained using D28 images and analysed via ImageJ [49]. Values were reported as mean ± SD (*n* = 6–9).

### 2.11. Hydroxyproline Quantification

Quantification of hydroxyproline within samples was determined to provide an indication of collagen content at the 28-day timepoint. Cultured TEVG samples (*n* = 6) from each group were analysed using a hydroxyproline assay kit (cat. no. MAK357, Sigma-Aldrich, USA). Samples were hydrolysed at 120°C for 1 hour in pressure-tight, screw-capped polypropylene vials with 100 µL of 10 M sodium hydroxide. Following alkaline hydrolysis, samples were cooled briefly on ice, prior to the addition of 100 *μ*L of 10 M hydrochloric acid to neutralise hydrolysates. Hydrolysates were then assayed in duplicate, with absorbance measured at 560 nm (ClarioStar Plus, BMG Labtech, Germany), and compared to a hydroxyproline standard as provided. Values were reported as mean ± SD (*n* = 6).

### 2.12. Statistical Analysis

Statistical analyses were performed using GraphPad Prism software (Dotmatics, USA). Measurements performed in replicate were reported as mean ± standard deviation (SD). Differences between groups were determined using one- and two-way analysis of variance (ANOVA) tests as appropriate, with values of *p* < 0.05 considered significant. Assessment of differences between groups was performed based on Tukey's multiple comparison tests, with a confidence level of 95% (*p* < 0.05) where symbols (*p* < 0.001^*∗∗∗*^, 0.001 < *p* < 0.01^*∗∗*^, 0.01 < *p* < 0.05^*∗*^) indicate significance in figures. Where applicable, interaction terms of the timepoints (TPs) and cell types (CTs) were determined.

## 3. Results

To explore the applicability of various cell sources in the biofabrication of TEVGs, we investigated commercially available human primary aortic SMCs as well as fetal ECFCs and MSCs isolated with high yields from the human full-term placenta following published protocols developed by the authors of [42]. Placenta-derived MSCs and ECFCs were particularly trialled here as novel cell sources for the generation of vascular tissue, with their translationally advantageous immunological profile identified as a potentially promising avenue for use in clinical applications. In this study, highly porous PCL scaffolds produced via MEW were utilised as substrates for tissue culture, the biomimetic mechanical properties of which we have characterised previously [31]. Defined populations of SMCs, MSCs, ECFCs, and a coculture of MSCs + ECFCs (1 : 5) were used for the culture of TEVGs for 28 days *in vitro.* The resultant constructs were analysed via histological, immunofluorescence, and SEM imaging, in combination with various biochemical assays throughout the culture period.

### 3.1. Cell Viability Assessment

**Figure 1:**
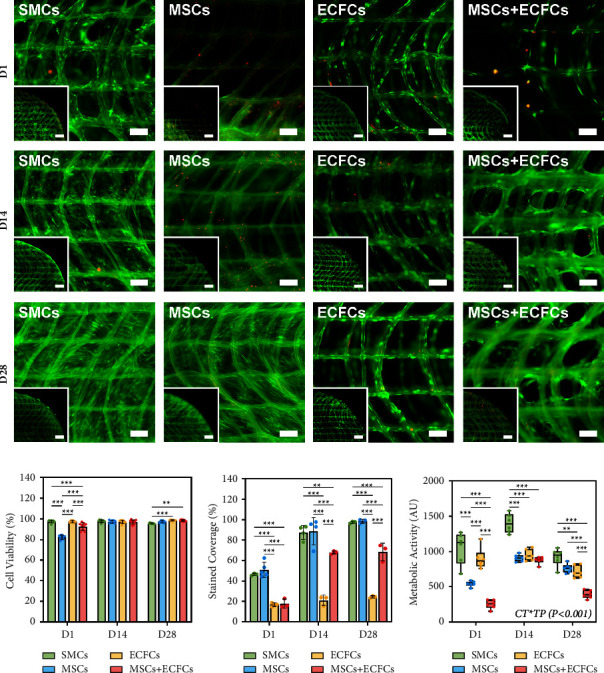
Cell viability and coverage of PCL scaffold-based TEVGs cultured with SMCs, MSCs, ECFCs, and MSCs + ECFCs (1 : 5) at days 1, 14, and 28. (a) Representative FDA/PI-stained micrographs showing live (green) and dead (red) cells, with inset images depicting quarters of biopsy punched samples. Primary scale bars: 100 *μ*m; inset scale bars: 500 *μ*m. (b) Quantified cell viability across timepoints reported as a percentage (%) of live to total cells, based on FDA/PI-stained samples (*n* = 3–6) analysed via ImageJ. (c) Stained coverage data determined as a percentage (%) area of live cells to the total area and analysed via ImageJ (*n* = 3–6). (d) Metabolic activity of samples (*n* = 6) across timepoints, assessed via PrestoBlue assay. The interaction term between timepoints (TP) and cell types (CT) reported with corresponding significance levels indicated. All values are reported as mean ± SD, where symbols (*p* < 0.001^*∗∗∗*^, 0.001 < *p* < 0.01^*∗∗*^, 0.01 < *p* < 0.05^*∗*^) indicate significance levels, based on ANOVA tests.

Cell viability was high (>90%) across timepoints for all groups, apart from the D1 MSC group, which recovered notably across subsequent timepoints (Figures 1(a) and 1(b)). Live and dead cell staining also enabled the determination of construct coverage based on the proportion of stained to the total area of analysed regions across multiple samples (Figure 1(c)). Cell attachment to the PCL fibres was evident at D1, with greater stained area in the SMC and MSC groups, compared to low coverage in the ECFC and MSC + ECFC groups. At D14, extensive growth and branching of SMCs and MSCs were evident, with coverage of the scaffold pores in excess of 85% in both groups. Significantly lower coverage was noted in the MSC + ECFC group, while ECFC coverage is very low, with cells remaining elongated along PCL fibres rather than spanning pores. By D28, complete coverage (>95%) was achieved by the SMCs and MSCs.

### 3.2. Metabolic Assays

While direct comparisons in metabolic activity between cell types are difficult to determine due to cell-specific baseline activity and growth rates, SMCs exhibited the greatest metabolic activity, with other cell groups statistically similar by D14 (Figure 1(d)). Increased metabolic activity was observed in all groups between D1 and D14, except the ECFCs which remained stable. From D14 to D28, a consistent downward trend in activity was observed across all groups, with cells potentially tending toward an ECM-producing, homeostatic state, as discussed subsequently, coinciding with results ascertained via histological staining.

### 3.3. SEM Imaging

**Figure 2:**
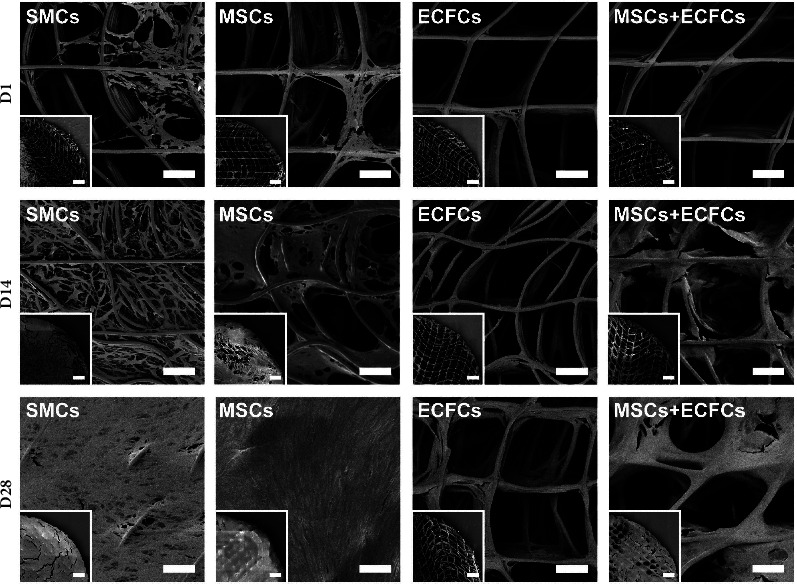
SEM images of TEVG constructs cultured from SMCs, MSCs, ECFCs, and MSCs + ECFCs (1 : 5) at day 1, 14, and 28 timepoints. Representative micrographs at 500x magnification, depicting TEVG samples, showing cell attachment, growth, and coverage of scaffold pores across timepoints for each of the cell groups cultured *in vitro*, with inset images depicting quarters of the 5 mm diameter biopsy punched scaffold samples at 55x magnification. Primary scale bars: 100 *μ*m; inset scale bars: 500 *μ*m.

MEW additive manufacturing enabled the production of highly porous, consistent scaffolds with biomimetic mechanical properties, as comprehensively characterised in our previous published work [31]. The scaffold design incorporated sinusoidal fibres to mimic the anisotropic mechanical properties of vascular tissue [50, 51]. Samples accurately replicated the intended G-code pattern designed with a 250 *μ*m pore spacing, with scaffolds exhibiting consistent spacing of 247 ± 17 *μ*m (*n* = 60). Furthermore, fibre diameter was measured to be 11.3 ± 0.7 *μ*m (*n* = 60), with scaffold thickness determined to be 396 ± 20 *μ*m (*n* = 60). Limited fibre misalignment was observed, with minor irregularities attributed to the accumulation of electrostatic charges causing repulsion of the polymer jet during extrusion, as noted in the literature [52]. SEM imaging of TEVG samples (Figure 2) provided qualitative indications of cellular growth, coverage, and distribution comparable to the fluorescence coverage data. D1 images emphasised the limited branching of ECFCs, while substantial attachment and preliminary spanning of pores by the SMCs and MSCs were evident. High magnification (1500x) images (Figure S2, Supplementary Data) provide further indications of the morphological differences in cell types at D1. While ECFCs remained close to the fibres throughout, by D14, the MSCs and SMCs exhibited near-complete coverage, with D28 coverage homogeneous across the constructs (Figure 2), noting that cracks visible within images are artefacts of processing. The coculture of MSCs + ECFCs exhibited considerable cellular distribution despite incomplete coverage across timepoints.

### 3.4. Phenotypic Characterisation

**Figure 3:**
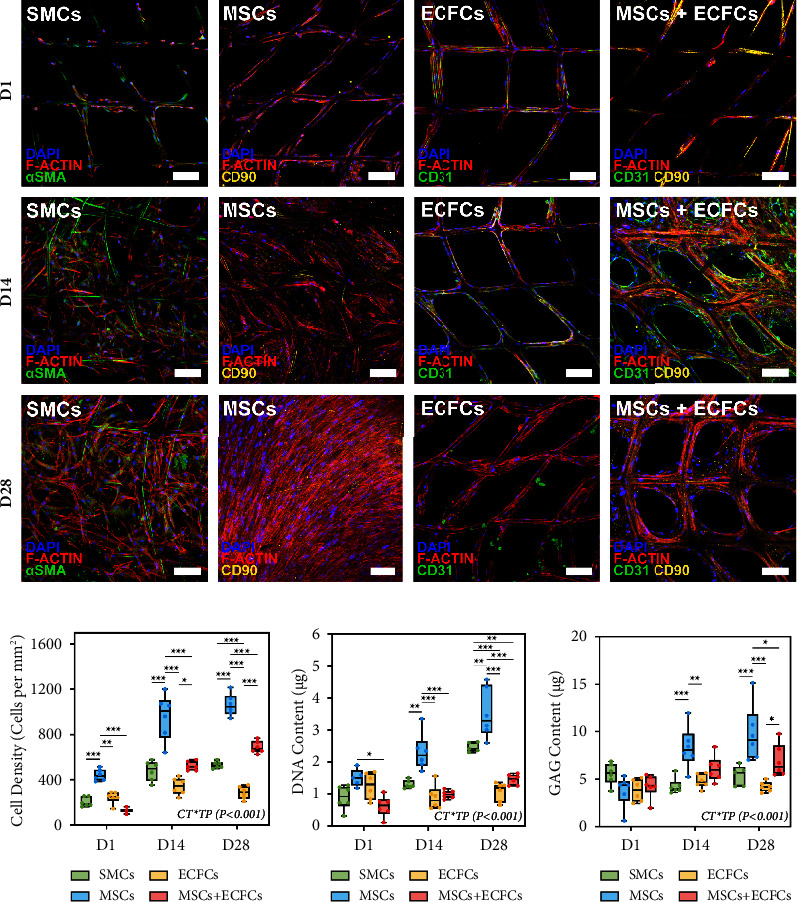
Phenotypic characterisation and biochemical assessment of TEVGs cultured from SMCs, MSCs, ECFCs, and MSCs + ECFCs (1:5) at day 1, 14, and 28 timepoints. (a) Representative micrographs of immunofluorescence-stained samples for each cell group; SMCs stained for expression of *α*SMA; MSCs stained for expression of CD90; and ECFCs stained for expression of CD31; all samples counterstained with DAPI and AF phalloidin 633. Scale bars: 100 *μ*m. (b) Density of nuclei per mm^2^ ascertained from multiple regions of samples (*n* = 3–6) from each group, analysed via ImageJ. (c) DNA content of samples (*n* = 5–6) assessed via PicoGreen assay. (d) GAG content of samples (*n* = 5–6) assessed via DMMB assay. All values are reported as mean ± SD, where symbols (*p* < 0.001^*∗∗∗*^, 0.001 < *p* < 0.01^*∗∗*^, 0.01 < *p* < 0.05^*∗*^) indicate significance levels, based on ANOVA tests. Interaction terms between timepoints (TP) and cell types (CT) reported with significance levels indicated.

Immunofluorescence staining (Figure 3) was performed to verify the expression of key phenotypic markers, with *α*SMA, CD90, and CD31 used to distinguish between SMCs, MSCs, and ECFCs, respectively. The different cell types were clearly discernible across the four groups, with DAPI and phalloidin counter-staining providing indications of nuclei density and F-actin distribution. The distribution and localisation of *α*SMA-positive SMCs across D1, D14, and D28 coincided strongly with the coverage results ascertained through viability and SEM imaging, demonstrating an invariable phenotype throughout the extended culture. Similarly, MSCs consistently expressed CD90 across timepoints, in both the MSC and MSC + ECFC groups. Notably, while sparsely distributed, CD31+ endothelial cells were observed in both groups containing ECFCs at D1 and D14; however, by D28, CD31 expression was negligible indicating a potential loss of phenotype, as discussed subsequently. Within the coculture, the ECFCs formed a monolayer covering of the MSCs and scaffold fibres in characteristic cobblestone patterns (Figure S3, Supplementary Data). Furthermore, cell densities (Figure 3(b)) quantified from DAPI-stained nuclei complemented the semiquantitative coverage measures ascertained via viability staining. With the exception of ECFCs, all groups exhibited significant increases (*p* < 0.001) in cell density from D1 to D28, with the MSCs most substantial (*p* < 0.001).

### 3.5. DNA and GAG Assays

Negligible differences in DNA content (Figure 3(c)) were determined between the SMC, MSC, and ECFC groups at D1, while the MSCs + ECFCs had notably lower DNA content indicating potentially reduced seeding efficiency. Only the MSCs exhibited a significant increase in DNA between D1 and D14 (*p* < 0.05), while other groups remained stable. Furthermore, DNA content in the MSC group was greater (*p* < 0.01) than other cell types at D14 and D28. Second to MSCs, the DNA content of TEVGs produced by SMCs increased (*p* < 0.001) from D14 and D28, at greater (*p* < 0.001) levels than both the ECFC and MSC + ECFC groups. Notably, no significant increase in DNA content was determined in the ECFC-only group, corresponding to the limited proliferation and metabolic activity, while a considerable increase (*p* < 0.05) in DNA was observed in the coculture from D1 to D28.

Only the MSCs (*p* < 0.01) and MSCs + ECFCs (*p* < 0.05) exhibited statistically significant accumulation of glycosaminoglycans (GAGs) across timepoints (Figure 3(c)). These results provide indications of the capacity of the fetal MSCs to produce GAGs within the four-week culture period, particularly when compared to the negligible increases in GAG content in the SMC group. GAG content in the ECFC group remained lower than all other groups and was stable between timepoints, as expected for the endothelial cells.

### 3.6. Histological Evaluation

**Figure 4:**
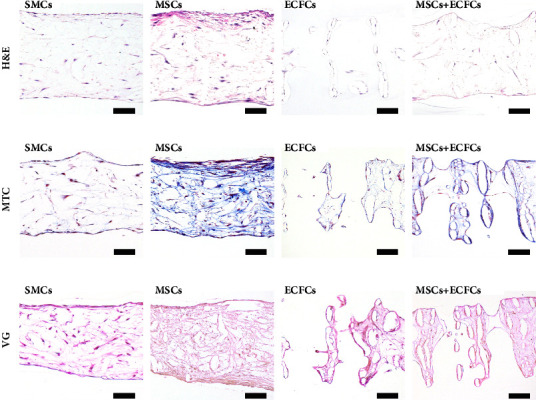
Histological characterisation of TEVG samples cultured from SMCs, MSCs, ECFCs, and MSCs + ECFCs (1 : 5) after 28-days *in vitro*. Representative micrographs of histologically stained paraffin-embedded cross sections (5 *μ*m) of tissue-engineered constructs, stained with (a) hematoxylin and eosin (H&E) for visualisation of cell nuclei distribution and ECM deposition; (b) Masson's trichrome (MTC) for visualisation of collagen and muscle components within the ECM; and (c) van Gieson (VG) for visualisation of collagen and elastin deposition within the ECM. Scale bars: 100 *μ*m.

Multiple histological stains were performed for qualitative analysis of ECM accumulation and morphology to assess the capacity of the cell types for *in vitro* tissue formation (Figure 4). At D28, substantially greater matrix deposition by MSCs was observed with ECM more densely distributed throughout the thickness of TEVG samples than other cell types (Figure 4(a)). Despite similar infiltration of the scaffolds by SMCs, comparatively less stained ECM was noted, with a similar stained matrix of MSC + ECFC samples, despite lower infiltration and coverage in the cocultured TEVG samples. The ECFC-seeded constructs exhibited negligible ECM staining, with limited nuclei present on the fibres. Masson's trichrome (MTC) staining revealed a significant accumulation of collagen and cytoplasmic muscle matrix components throughout the thickness of the TEVG samples, particularly in comparison to other groups (Figure 4(b)). Notably, layer formation was observed within the MSC group, which was not observed in any other groups. The MSC + ECFC group exhibited considerable staining for collagen components, despite lower coverage throughout the thickness. Despite the substantial accumulation of collagen and muscle components by the MSCs, stained elastin was negligible across all groups in van Gieson's (VG) staining (Figure 4(c)). Despite this, VG staining provided further indication of the abundant collagen deposition by MSCs and the distribution of components within and around the PCL scaffold substrates. ECM deposition within TEVG sample sections was also assessed across earlier timepoints, with the progressive accumulation of matrix components evident (Figures S4–S6, Supplementary Data).

### 3.7. Collagen Staining and Quantification

**Figure 5:**
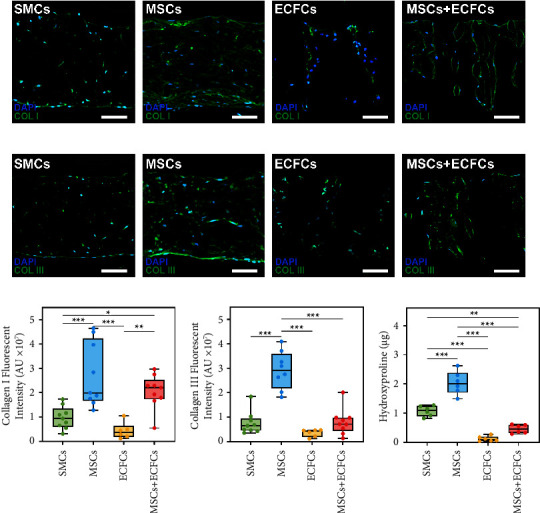
Representative micrographs of sectioned samples (5 *μ*m) of TEVGs cultured from SMCs, MSCs, ECFCs, and MSCs + ECFCs (1 : 5) after 28 days and immunostained for visualisation of collagen components. Representative sections immunostained for visualisation of (a) collagen I (COL I) (green) and (b) collagen III (COL III) (green) and counterstained with DAPI (blue). Scale bars: 100 *μ*m. Integrated fluorescence intensities of stained (c) collagen I and (d) collagen III at D28 and analysed via ImageJ. (e) Hydroxyproline content of D28 samples (*n* = 6) as an indicator of collagen synthesis. All values reported as mean ± SD (*n* = 6), where symbols (*p* < 0.001^*∗∗∗*^, 0.001 < *p* < 0.01^*∗∗*^, and 0.01 < *p* <  0.05^*∗*^) indicate significance levels, based on ANOVA tests.

At D28, immunofluorescence staining of collagen I (Figure 5(a)) and collagen III (Figure 5(b)) indicated extensive collagen accumulation in MSC-seeded samples. Morphologically, TEVG samples cultured with MSCs had the densest accumulation of collagen I, supported by the highest fluorescence intensity, followed by MSCs + ECFCs, SMCs, and ECFCs in decreasing order. Collagen III in the MSC group was less dense than collagen I, though still had significantly greater (*p* < 0.001) fluorescence intensity than other groups. The distribution of collagen I and III in the SMC group appeared morphologically similar, while the MSC + ECFC and ECFC sections exhibited minimal collagen III in the limited ECM produced. Quantification of hydroxyproline content (Figure 5(e)) coincided with histology and immunostaining. The MSCs exhibited the highest hydroxyproline content, significantly greater (*p* < 0.001) than other cells, followed by SMCs, ECFCs, and the coculture with very limited hydroxyproline detected.

## 4. Discussion

In this investigation, comparative analyses of the regenerative potential of various cell types suitable for the biofabrication of small-diameter vascular grafts have been presented. *In vitro* culture of TEVGs using SMCs, MSCs, ECFCs, and a combination of MSCs + ECFCs was performed over an extended four-week period utilising PCL scaffold substrates produced via MEW. Characterisation of differences in cellular attachment, proliferation, ECM deposition, and collagen production was ascertained via SEM and histological and immunofluorescence imaging, in combination with biochemical quantification of DNA, GAG, and hydroxyproline content.

Throughout the 28-day culture period, TEVGs maintained high cell viability (Figure 1) across groups validating construct compatibility with various cell types, consistent with our previous studies [31]. Extensive branching was exhibited by SMCs and MSCs, with near-complete infiltration and coverage of scaffolds by D28. Contrastingly, ECFC coverage was low as cells covered the PCL fibres without branching. This was expected given the propensity of endothelial cell types to form monolayers [53]. Between the D1 and D14 timepoints, significant increases in metabolic activity of the SMC, MSC, and coculture groups were noted, with enhanced proliferation coinciding with increases in cell density between initial timepoints (Figure 3). In contrast, ECFC activity was stable throughout, coinciding with limited growth. Interestingly, between later timepoints, each group exhibited reduced metabolism, suggesting a trend toward nonproliferative and ECM-producing states. SEM imaging supported the cell attachment and branching observations made, with significant growth and distribution of the SMCs and MSCs (Figure 2). By D28, each of these groups exhibited complete coverage with sheet-like morphology over the entirety of the samples, with histological staining validating the infiltration of cells and ECM throughout the thickness of the constructs.

Additionally, while the morphological and mechanical properties of the scaffolds have been comprehensively characterised previously in our work [31], SEM analysis prior to scaffold seeding enabled validation of the reproducibility of the MEW process utilised. Minimal deviation in the laydown of MEW fibres was observed, with uniform spacing and negligible variation in fibre diameter (coefficient of variation = 6.1%). These measures demonstrate the high replicability achieved in our studies, emphasising the suitability for scalable production of TEVG substrates with defined architecture and biomimetic properties.

Quantification of DNA and GAG accumulation (Figure 3) throughout the tissue culture period reinforced the outcomes discussed thus far. Most notably, despite increases in DNA, accumulation of GAGs by SMCs was minimal, with MSCs significantly out-performing all cell types, with the greatest accumulation of GAGs within TEVG samples. ECFC content was consistently low, with negligible differences in DNA or GAG across timepoints. MSCs + ECFCs exhibited reasonable production of GAGs after four weeks, with a similar trend in DNA, albeit significantly less overall than MSCs alone. These results were particularly notable given the low ratio of MSCs to ECFCs. While it may be inferred that MSCs in the coculture contributed most substantially to the DNA and GAG accumulation, the successful culture of these two cell populations in the formation of TEVGs is highly relevant for future studies.

Immunofluorescence staining performed enabled visualisation of cell-specific markers for confirmation of phenotype expression (Figure 3). *α*SMA, CD90, and CD31 were used to distinguish between SMCs, MSCs, and ECFCs, respectively, with cell types clearly discernible across the four groups at D1. Progressive cell growth was evident through immunostaining, in the SMC and MSC groups, with significant positive expression of *α*SMA and CD90, respectively, from D1 to D28 demonstrating an invariable phenotype of these cell types throughout. Comparatively, while positive expression of CD31 by ECFCs was observed at D1 and D14, limited expression was noted at D28. The nonexpression of CD31 was consistent in both ECFC groups, suggesting the ECFCs may have undergone phenotype variation by D28 due to their plasticity, with the static culture conditions a potential influencing factor. The loss of CD31 expression after 4 weeks requires further investigation in future work, to overcome such limitations. Of particular note is the planned inclusion of dynamic flow conditions, noted throughout the literature to improve vascular endothelialisation and ECFC survival *in vitro,* by simulating the physiological biomechanical environment [54].

Despite this, the successful culture of these specific endothelial cells at high viability throughout remains noteworthy; particularly for future studies whereby ECFCs may be seeded onto precultured TEVGs for endothelialisation as we have demonstrated with alterative endothelial cells previously [31]. This may offer considerable upside, given that throughout the initial 14-day period, ECFCs were observed to form a monolayer surrounding the MSCs in coculture and scaffold fibres alone, with strong marker expression exhibiting an identifiable cobblestone pattern (Figure S3, Supplementary Data). Through the inclusion of ECFCs after extended MSC-culture, the formation of a confluent luminal lining for enhanced biological performance may be achieved, as shown with endothelial cells throughout the literature [55, 56].

It may be surmised from these data that of the cell types utilised to culture TEVGs herein, MSCs and SMCs exhibited the most notable growth and coverage, demonstrating extensive branching and infiltration of the porous scaffolds, consistent with findings within our previous studies [31]. These observations were supported by histological assessment of sectioned samples enabling visualisation of ECM deposition and cellular infiltration throughout the thickness of the TEVG samples (Figure 4). While the production of ECM components within all groups was assessed across timepoints (Figures S4–S6, Supplementary Data), morphological assessment of D28 samples indicated the greatest ECM production within the MSC-cultured samples. This was particularly evident when MSC groups were compared to ECFC and MSC + ECFC samples, where numerous gaps in tissue formation were present. The SMCs exhibited some ECM formation by D28, though the density was considerably lower, with MTC staining revealing limited collagen deposition by SMCs. The reduced performance of SMCs during the 28-day period is consistent with reports which indicate that for the production of vascular media, SMCs require a long *in vitro* process [57]. Hence, it may be suggested that while MSCs enabled extensive collagen and muscle fibril deposition, a longer culture period may be required to achieve comparable results with SMCs.

Immunofluorescence staining of collagen I and III (Figure 5) strongly supported these observations, with quantification further emphasising the enhanced performance of the MSCs throughout this study. While the fluorescence intensities determined remain semiquantitative measures, when considered in combination with the observations made regarding the distribution of collagen throughout the matrix of each of the groups, the performance of the MSCs in comparison to other groups was emphatically greater. Staining of collagen I was slightly more evenly distributed within the D28 MSC groups in comparison to collagen III, though statistical comparisons between the data cannot be made directly due to differences in staining concentrations and intensities. Within all alternative culture groups, very limited expression of collagen I and III was observed at D28, corresponding with the reduced ECM deposition observed through histology. Morphologically, results were consistent in each group, with the SMC group demonstrating considerable infiltration throughout the TEVGs, despite the reduced density of collagen produced, while the groups containing ECFCs remained sparse, with minimal branching or matrix accumulation.

These findings related to the production of collagen specifically within the ECM were reflected in the quantification of hydroxyproline at D28 (Figure 5(e)). As an amino acid specifically associated with collagen, quantification of hydroxyproline provided a reliable indication of collagen accumulation in the assessment of collagen synthesis within TEVGs. Thus, quantification at D28 indicated that the accumulation of hydroxyproline was most significant within the MSC group, with content twice as great as the SMC group. Negligible hydroxyproline content was determined within the ECFC group, at more than 15 times less than the MSCs alone. Such results were not unexpected, with ECFCs a cell type not commonly associated with substantial tissue formation. In comparison, while the MSC + ECFC group exhibited hydroxyproline accumulation 4.4 times less than within the MSC-only group, the coculture exhibited reasonable hydroxyproline accumulation at D28 considering the relative seeding density of MSCs to ECFCs. Overall, the data provide a number of interesting points for consideration and inclusion in future studies.

Despite SMCs being the most frequently used in the biofabrication of TEVGs [15, 57–61], the results of this study align with the limitations of SMCs as reported by others, suggesting that SMCs are potentially less suited than MSCs given the extended culture time reported for SMCs to achieve substantial ECM deposition [57]. Thus, as the results ascertained herein indicate, MSCs represent a potentially superior cell type, which can be readily isolated and expanded *in vitro* as we have demonstrated previously from placental tissue [42, 44]. It must be noted that while MSCs are not the main cell types present in native vasculature, their relevance and suitability for use in vascular tissue-engineering remain high, owing to their scalability, regenerative applicability, and suitability for the production of relevant ECM [62–64]. In line with published studies, further research into the differentiation of MSCs is required to determine whether phenotype changes occur [65], particularly when cultured under dynamic conditions toward vascular phenotypes, as observed in the literature [63].

Furthermore, the immunogenicity of fetal stem cell sources, such as those utilised in this study, is often reported as being of low or negligible impact [66]. However, it has been shown that upon implantation, immunogenicity may increase due to the immune response within the recipient's native tissue, particularly when inflamed or damaged [66]. Such concerns must be addressed if TEVGs are to be implanted as cellular constructs, with strategies to mitigate increased immunogenicity including the local delivery of immunosuppressive drugs or ongoing immunomodulatory treatments for the promotion of an anti-inflammatory microenvironment [67]. Additionally, autologous or treated matched allogenic stem cell types may be used to overcome these issues. However, it has become well-established in the field that decellularized treatment options that exhibit physiological tissue matrix and have limited immunogenicity represent an ideal solution for the clinical translation of TEVGs [67–69]. As off-the-shelf products, acellular TEVGs mitigate many concerns associated with the *in vivo* immune response and may be coupled with autologous endothelial cell types if required [67]. Hence, in ongoing and future work associated with this study, the investigation into the biofabrication of decellularized TEVGs is required.

Thus, methods of cellular removal via decellularization strengthen the applicability of MSCs in the biofabrication of TEVGs. Through the implementation of processes frequently applied to SMC-derived grafts [61, 65, 70], the dense accumulation of ECM components *in vitro* may be facilitated prior to the removal of cellular components, leaving a highly relevant ECM-free of cell-specific components, mitigating immunogenic concerns. Decellularization is a postprocessing methodology toward the mitigation of immunogenicity, broadening the range of applicable cell sources and types that may be utilised for TEVG biofabrication [71], as demonstrated in various studies [61, 65, 72–74]. Through the application of decellularization protocols, the ECM deposited by specified cell types remains as the remnant bioactive component, which may be optimised for *in vivo* integration and recellularization within the host tissue. Off-the-shelf TEVGs are highly desirable for clinical applicability, with decellularized TEVGs representing a potentially readily available alternative to current clinical grafting options, whereby acellular grafts may be further combined with patient-specific endothelial cell types for enhanced acceptance and patency [68].

A potential alternative stem cell type to those studied herein, suitable for use in vascular regeneration, is induced pluripotent stem cells (iPSCs) [13], which represent a quasi-unlimited cell source with high differentiation capacity [4, 75] and immense scale-up potential [76]. iPSCs exhibit greater availability and superior proliferative ability than adult primary and stem cell sources, while possessing extensive multilineage differentiation capability [16]. Furthermore, iPSCs have been identified to produce ECM components with higher yields than autologous cells [11, 16, 77]. Specific to vascular biofabrication, Luo et al. [16] successfully utilised human iPSC-derived cells on PGA scaffolds, which produced a collagen-rich ECM [78]. Patient-specific iPSCs may be isolated from a multitude of accessible tissue sources, such as peripheral blood samples or skin biopsies, overcoming immunological and ethical considerations [27]. Despite the numerous benefits of iPSCs, limitations associated with expansion time and scalability require optimisation for efficient expansion of iPSCs prior to clinical application in TEVGs [27, 28, 76]. Alternatively, xenogeneic sources of differentiated cell lines have demonstrated scalable *in vitro* production capacity, though present challenges associated with applicability to human studies and immunogenicity, requiring decellularization treatments as previously introduced, prior to implantation [20].

In a review covering the use of ECFCs in vascular tissue engineering, Banno and Yoder [79] detail the clonogenic and proliferative potential of ECFCs in comparison to adult sources. Furthermore, it was identified that the generation of patient-specific hiPSC-derived ECFCs may be suitable to overcome limitations surrounding the availability of autologous sources in certain patient populations [79]. This is one consideration for the translation of placental ECFCs, with clinical scale-up approaches required; hence, many studies have investigated umbilical and peripheral blood-derived ECFCs [54, 80, 81]. Specifically, Kraus et al. [54] ascertained that ECFCs derived from human peripheral blood were able to be differentiated toward an arterial phenotype. Additionally, it was ascertained that the application of dynamic flow resulted in ECFC elongation, with significant differences in gene expression observed in comparison to adult endothelial cell populations, with high levels of antithrombotic genes [54].

Thus, while optimisation of ECFCs use in the biofabrication of vascular grafts is required, the results of this study, in particular the compatibility of ECFCs with our scaffolds and cultured MSCs constructs, warrant continued investigation. In future studies, we plan to include ECFCs after extended MSC culture to facilitate endothelialisation through the formation of a monolayer coating of the TEVG lumen, as we have demonstrated previously [31]. Limitations associated with the methodologies of this investigation include the use of static culture conditions, whereby dynamic culture practices remain specifically relevant for enhanced production of robust vascular tissue, as evident throughout the literature [16, 82]. As such, to extend upon the findings determined herein, future studies incorporating dynamic tissue culture conditions are required to determine the potential enhancement of ECM deposition toward improved graft performance. Through stimulation of the culture environment, the production of TEVGs comprised of MSCs and endothelialised with ECFCs may be enhanced for long-term assessment and preclinical investigations.

Such improvements have been described in the literature, whereby adult sources of MSCs and ECFCs have been utilised in a two-stage coculture, demonstrating positive results toward the biofabrication of multilayered, multicellular TEVGs [63]. Notably, the use of a perfused bioreactor has been reported to enable successful ECFC luminal surface coating of an MSC-based TEVG, a coculture methodology that may be incorporated in future studies to complement the findings described herein, with the aim of improving the biofabrication of TEVGs with dense matrix accumulation by MSCs, and confluent ECFC lining. Aside from the use of a bioreactor culture, the main difference in these studies remains in the use of adult bone-marrow-derived MSCs and adult cord blood ECFCs, as described by Pennings et al. [63]. Thus, while the *in vitro* results reported are comparable to those obtained herein, with extensive cell growth within scaffold substrates coinciding with vascular tissue formation over time, the use of pure fetal MSCs and ECFCs derived from placental tissue in this study represents a novel application of this cell type. Furthermore, through the use of the established high-yield isolation strategy, we have demonstrated the superior regenerative potential of these readily available stem cell types in comparison to frequently used SMCs, toward the manufacture of TEVGs. Improvements toward incorporation of dynamic biofabrication methods, in line with those described in the literature [63, 83, 84], are planned for inclusion in future work to extend upon the findings of this investigation, utilising MSCs alongside ECFCs for the biofabrication of biomimetic grafts to be tested in preclinical studies.

## 5. Conclusion

Throughout this investigation, comparative analyses of the regenerative potential of novel MSC and ECFC sources for the biofabrication of TEVGs provided positive results. Extended culture of adult SMCs alongside fetal MSCs and ECFCs seeded onto highly porous PCL scaffold substrates enabled characterisation of differences in cellular attachment, proliferation, and ECM deposition. Most notably, TEVGs cultured with MSCs exhibited extensively greater biological performance than other cell types, with enhanced DNA, GAG, and collagen accumulation, in combination with substantial tissue growth discernible through histological, immunofluorescence, and SEM imaging. While SMCs infiltrated and covered scaffold substrates completely after 28 days, deposition of collagen and matrix components was limited. Preliminary endothelialisation of constructs was achieved using ECFCs, though optimisation in future work is required for successful integration *in vivo.* Of cells investigated, MSCs were identified as the preferred cell source for tissue generation within the porous scaffolds, while the inclusion of ECFCs provided results which warrant investigation in future work. Overall, these findings demonstrate the regenerative potential of cells sourced from placental tissue, in the biofabrication of grafts for use in vascular bypassing procedures.

## Data Availability

The data that support the findings of this study are available upon reasonable request.

## Additional Points

*Statement of Significance*. Through this comparative investigation into various human cell types in the development of biological vascular grafts for small-diameter bypassing, this study aimed to identify a novel application of pure fetal stem cells with superior biological performance in tissue engineering. In comparison to adult smooth muscle cells, fetal stem cells demonstrated superior performance, producing tissue with vital extracellular matrix components, while placental endothelial cells showed potential for endothelialisation. Through the translation of stem cell technologies in tissue-engineering applications, small-diameter lab-grown vascular grafts with nonimmunogenic properties are expected to improve outcomes for patients with cardiovascular disease.

## Supplementary Materials

Supplementary Materials The supplementary material file provides additional data depicting (Figure S1) diagrammatic study methods for tissue culture of MEW scaffolds; (Figure S2) high magnification SEM images of MEW scaffold cell attachment; (Figure S3) high magnification immunofluorescence-stained images of cell phenotype markers; and (Figure S4–S6) histological staining across timepoints with H&E, Masson's trichrome, and van Gieson stains.
