# Supplementary material for: Harnessing the Regenerative Potential of Fetal Mesenchymal Stem Cells and Endothelial Colony-Forming Cells in the Biofabrication of Tissue-Engineered Vascular Grafts (TEVGs)
Source: J Tissue Eng Regen Med. 2024 Jun 12;2024:8707377. doi: 10.1155/2024/8707377 (PMC11919237; doi:10.1155/2024/8707377)
Supplement: Supplementary Materials — The supplementary material file provides additional data depicting (Figure S1) diagrammatic study methods for tissue culture of MEW scaffolds; (Figure S2) high magnification SEM images of MEW scaffold cell attachment; (Figure S3) high magnification immunofluorescence-stained images of cell phenotype markers; and (Figure S4–S6) histological staining across timepoints with H&E, Masson's trichrome, and van Gieson stains. [file 8707377.f1.pdf]

SUPPLEMENTARY DATA

Harnessing the regenerative potential of fetal mesenchymal stem cells and endothelial colony-forming cells in the biofabrication of tissue-engineered vascular grafts (TEVGs)

Angus Weekes [a, b, c], Joanna M. Wasielewska [c, d], Nigel Pinto [c, e], Jason Jenkins [c, e], Zhiyong Li [a, b], Jatin Patel [f], Travis J. Klein [a b,], Christoph Meinert [a, b, c,*]*

Queensland University of Technology (QUT), Brisbane, Australia
Herston Biofabrication Institute, Brisbane, Australia

Diagrammatic representation of study methods for tissue culture of MEW scaffolds

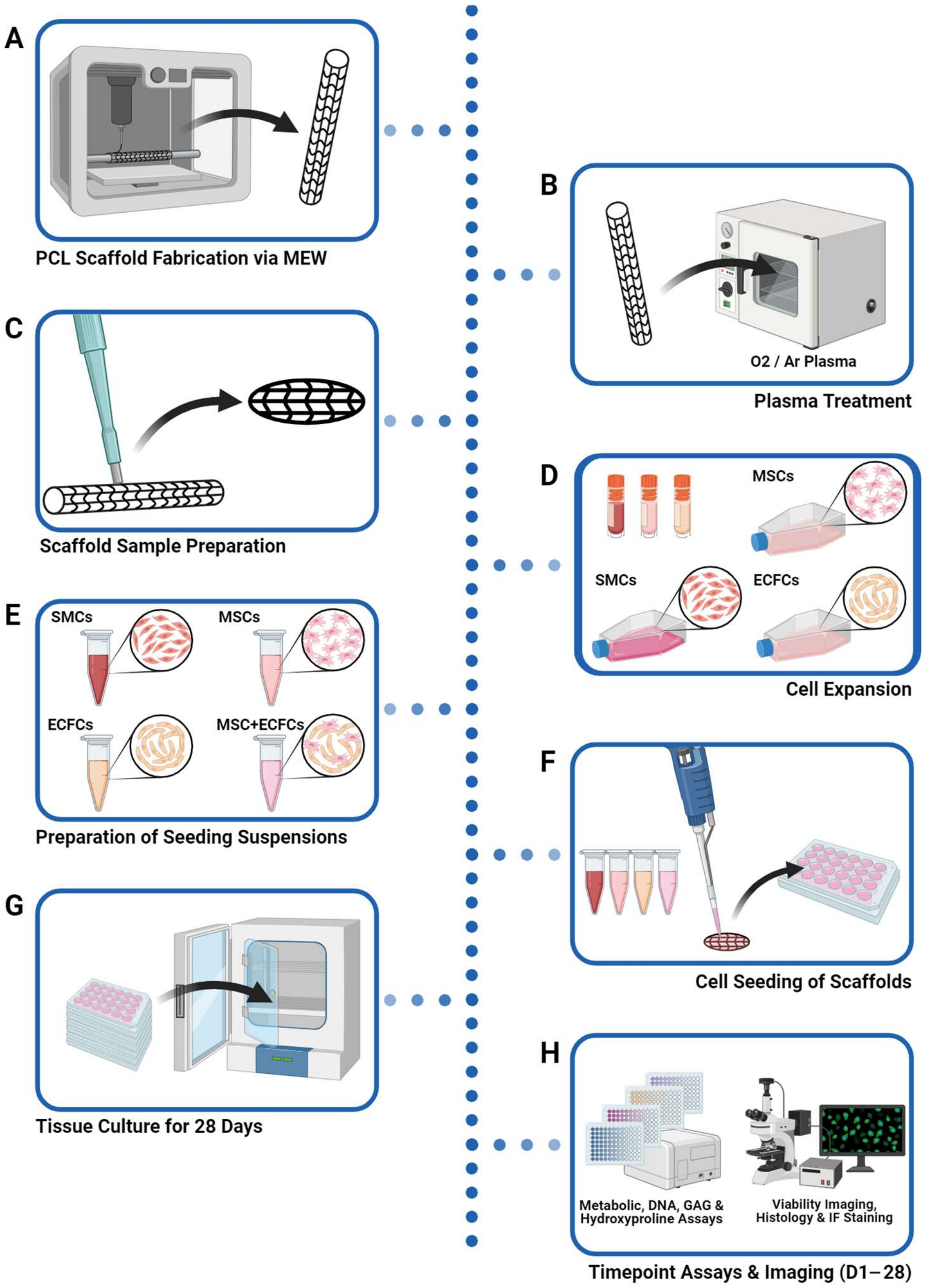

Figure S1. Diagrammatic representation of experimental method stages in the biofabrication of vascular tissue constructs from MEW scaffolds. (A) Fabrication of PCL scaffolds via MEW additive manufacturing. (B) Plasma treatment (O_2 / Ar) of scaffolds for induced hydrophilicity. (C) Collection of 5 mm biopsy samples of scaffolds suitable for tissue culture. (D) Expansion and (E) preparation of SMCs, MSCs, ECFCs and MSCs+ECFCs (1:5 co-culture) seeding suspensions prior to (F) seeding of PCL scaffolds for (G) extended culture *in vitro*. (H) Timepoint assays performed included metabolic activity, DNA, GAG and hydroxyproline assessment, as well as FDA/PI cell viability imaging across timepoints, with fixed sample immunostaining, fluorescence imaging and histology performed. Figure created with BioRender.

Scanning electron microscopy (SEM) – 1500X magnification

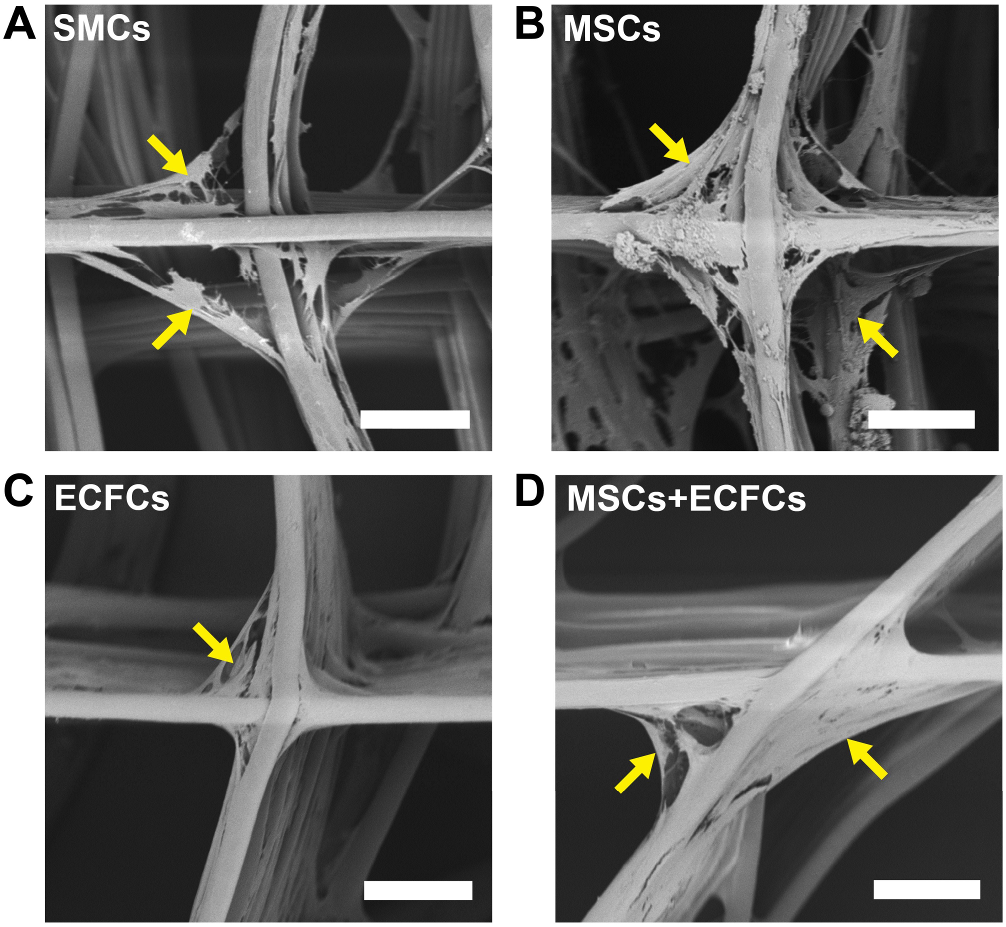

Figure S2. SEM images of cellular attachment on PCL scaffold substrate samples at D1. Representative micrographs at high (1500X) magnification of (A) SMCs, (B) MSCs, (C) ECFCs, and (D) MSC+ECFCs (1:5) depicting differences in cell attachment on scaffold fibres cultured *in vitro*. Scale bars: 50 μm. Arrows provided to indicate cell attachment to PCL fibres.

High magnification immunofluoresence-stained images of cell phenotype markers

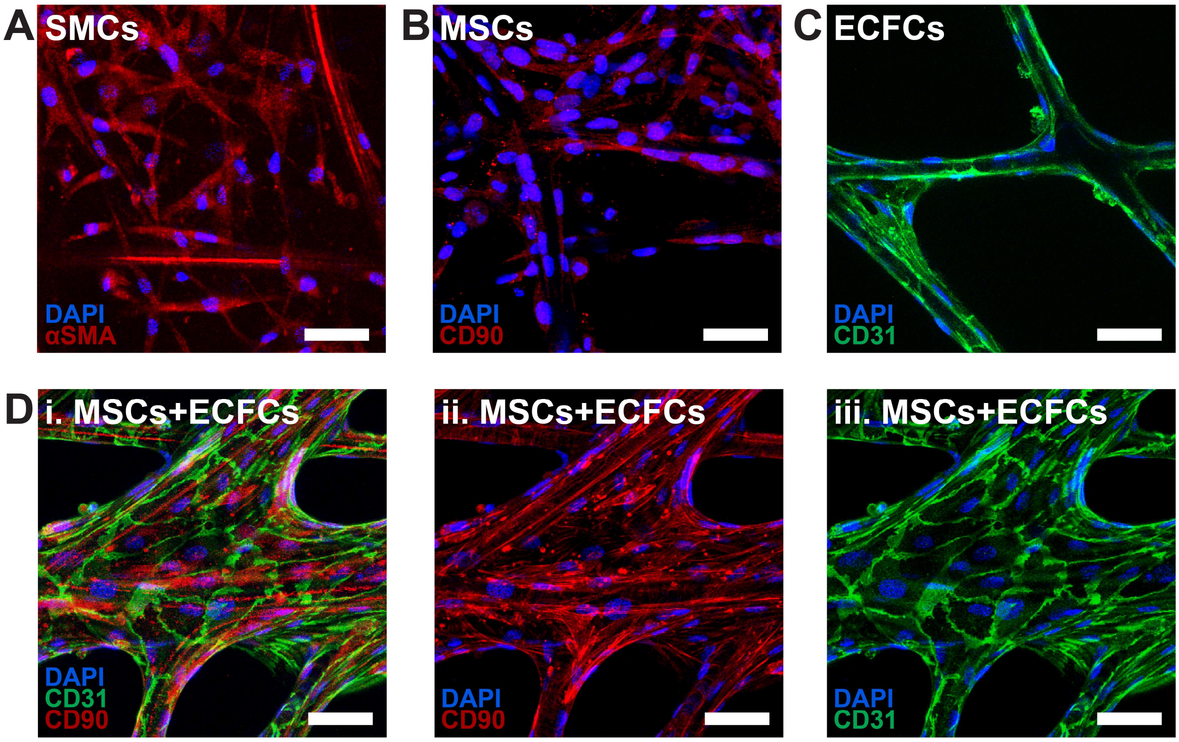

Figure S3. CLSM images of immunostained cell-seeded scaffold TEVGs showing characteristic cellular phenotype marker expression at D14. Representative regions of interest from scaffolds seeded with (A) SMCs, stained for expression of αSMA; (B) MSCs, stained for expression of CD90; (C) ECFCs stained for expression of CD31; and (D) MSCs+ECFCs stained for expression of CD90 and CD31, respectively; all samples counterstained with DAPI. Scale bars: 50 µm.

Histological staining across timepoints – hematoxylin and eosin (H&E)

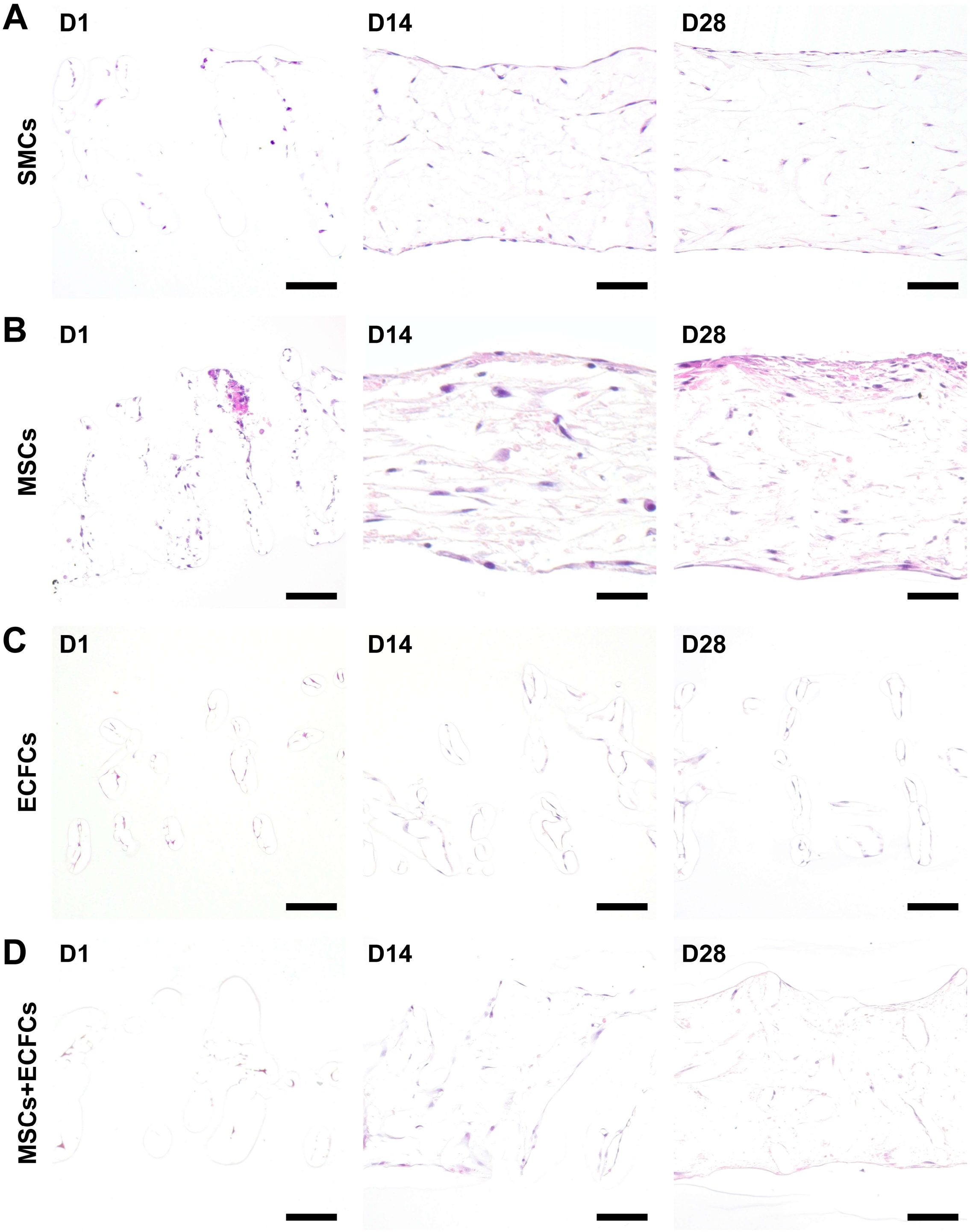

Figure S4. Representative hematoxylin and eosin (H&E) stained histological sections (5 μm) of PCL scaffolds cultured *in vitro*. H&E-stained sections cultured with (A) SMCs, (B) MSCs, (C) ECFCs, and (D) MSCs+ECFCs, across D1, D14 and D28 timepoints. Scale bars: 100 μm.

Histological staining across timepoints – Masson’s trichrome (MTC)

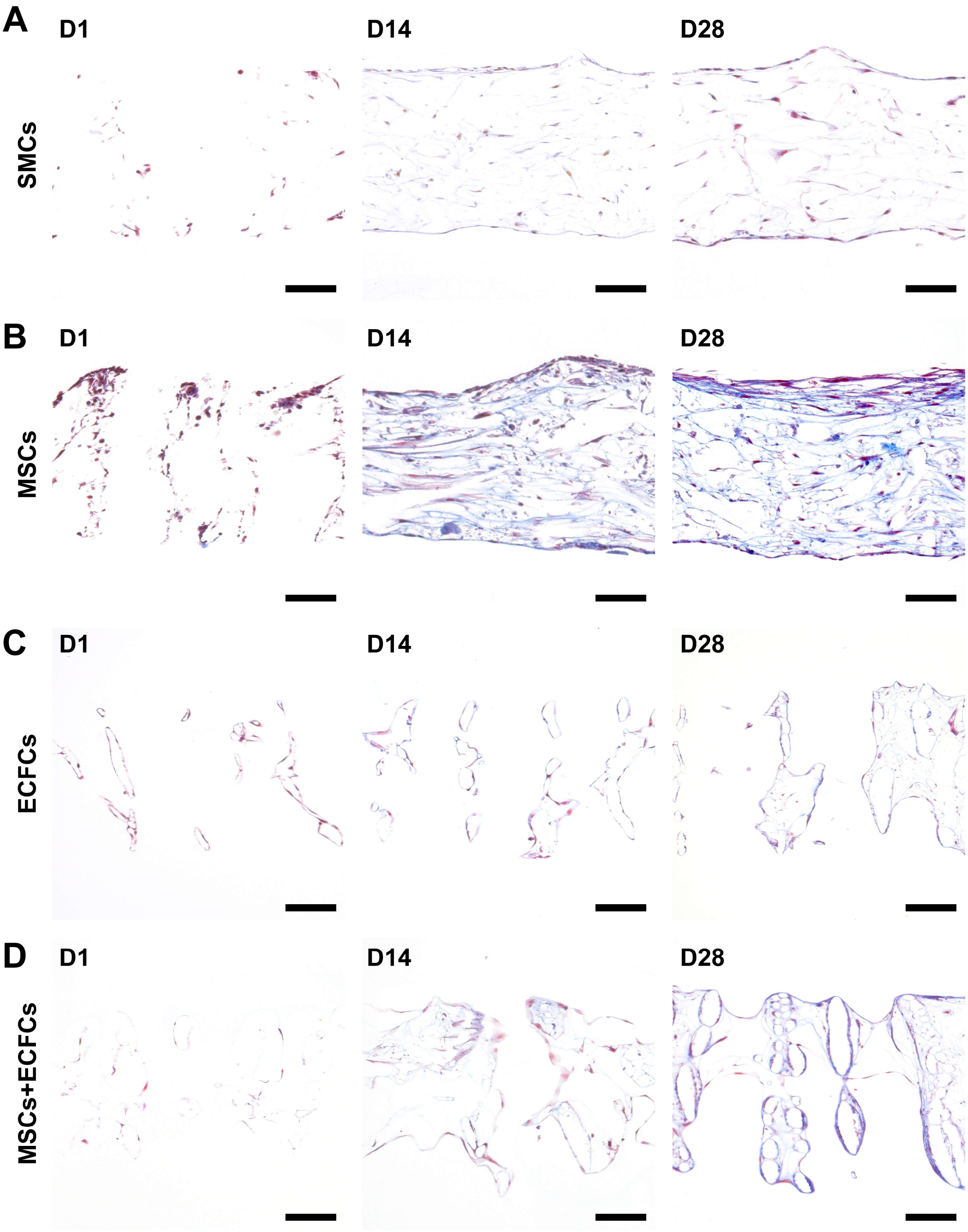

Figure S5. Representative Masson’s trichrome (MTC) stained histological sections (5 µm) of PCL scaffolds cultured *in vitro*. MTC-stained sections cultured with (A) SMCs, (B) MSCs, (C) ECFCs, and (D) MSCs+ECFCs, across D1, D14 and D28 timepoints. Scale bars: 100 µm.

Histological staining across timepoints –van Gieson (VG)

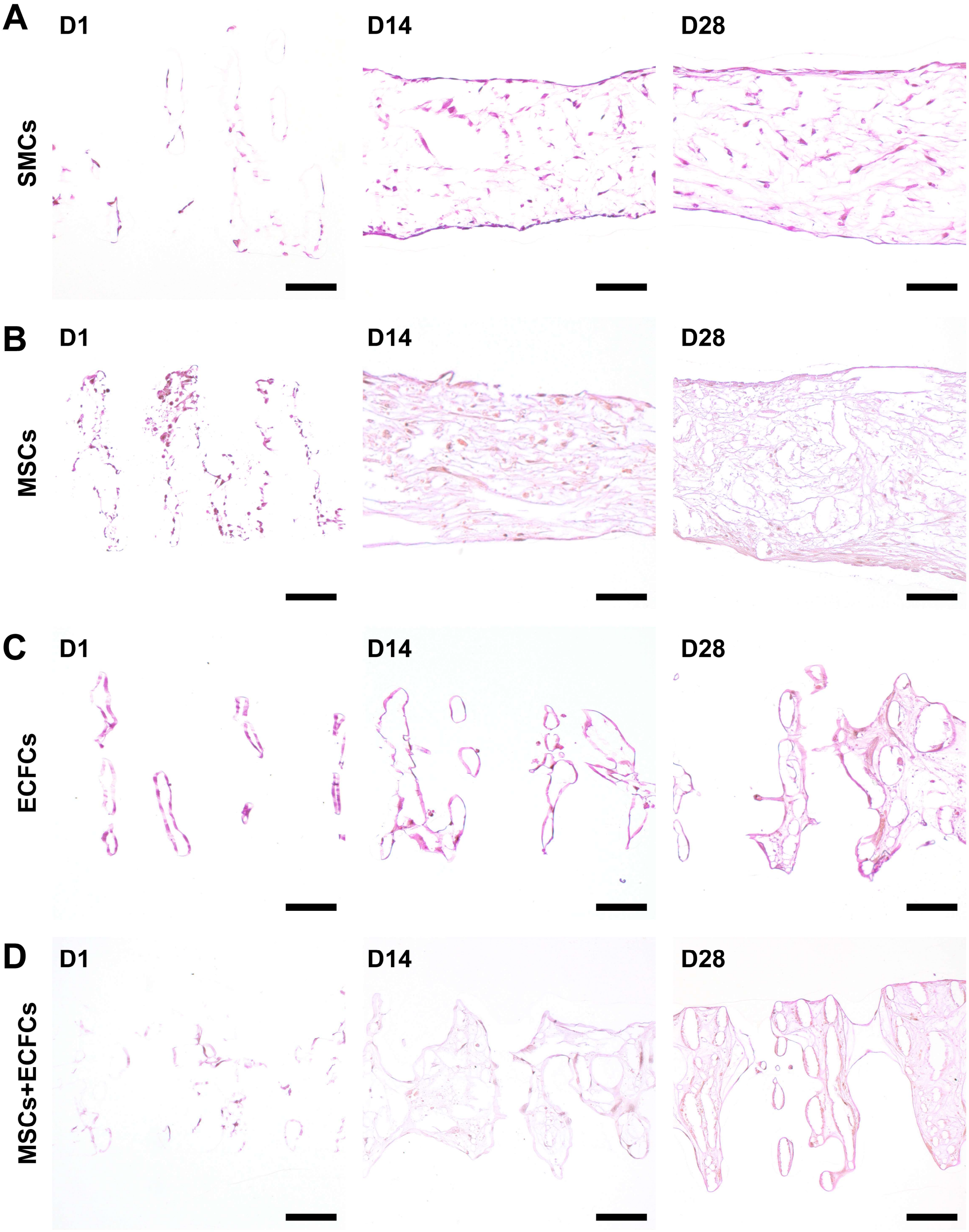

Figure S6. Representative van Gieson (VG) stained histological sections (5 µm) of PCL scaffolds cultured *in vitro.* VG-stained sections cultured with (A) SMCs, (B) MSCs, (C) ECFCs, and (D) MSCs+ECFCs, across D1, D14 and D28 timepoints. Scale bars: 100 µm.
